# Monoclonal Antibody-Based Colorimetric Lateral Flow Immunoassay for the Detection of Pyridaben in the Environment

**DOI:** 10.3390/bios13050545

**Published:** 2023-05-13

**Authors:** He Chen, Hao Liu, Yanran Ji, Zekun Sha, Li An, Meng Li, Di Zhang, Xujin Wu, Xiude Hua

**Affiliations:** 1 Institute of Quality Standard and Testing Technology for Agro-Products, Henan Academy of Agricultural Sciences, Zhengzhou 450002, China; 2 Key Laboratory of Grain Quality and Safety and Testing Henan Province, Zhengzhou 450002, China; 3 School of Life Sciences, Henan University, Kaifeng 475004, China; 4 College of Plant Protection, Nanjing Agricultural University, Nanjing 210095, China

**Keywords:** pyridaben, monoclonal antibody, colorimetric lateral flow immunoassay, enzyme-linked immunosorbent assay, on-site detection

## Abstract

Pyridaben, a broad-spectrum pyridazinone acaricide that is widely used in agricultural production, can induce neurotoxicity and reproductive abnormalities, and is highly toxic to aquatic organisms. In this study, a pyridaben hapten was synthesized and used to prepare monoclonal antibodies (mAbs), among which 6E3G8D7 showed the highest sensitivity in indirect competitive enzyme-linked immunosorbent assay, with a 50% inhibitory concentration (IC_50_) of 3.49 ng mL^−1^. The mAb, 6E3G8D7, was further applied to a gold nanoparticle-based colorimetric lateral flow immunoassay (CLFIA) for pyridaben detection, according to the signal intensity ratio of the test line to the control line, which showed a visual limit of detection of 5 ng mL^−1^. The CLFIA also showed high specificity and achieved excellent accuracy in different matrices. In addition, the amounts of pyridaben in blind samples detected by the CLFIA, were consistent with high-performance liquid chromatography. Therefore, the developed CLFIA is considered a promising, reliable, and portable method for pyridaben on-site detection in agro-products and environmental samples.

## 1. Introduction

Pyridaben, 2-*tert*-butyl-5-[(4-*tert*-butylphenyl)methylsulfanyl]-4-chloropyridazin-3-one, is a broad-spectrum, contact-killing pyridazinone agro-chemical acaricide that is mainly used to control spider mite and panonychus mite eggs, larvae, and adults [1,2]. The mechanism of action is the inhibition of glutamine dehydrogenase synthesis in the chromosome of the electron transport system [3,4]. Pyridaben is widely used in China, the European Union (EU), the USA, and other countries, and its residues have been detected in cabbage, fruits, and paddy water [2,5]. Previous studies have demonstrated that pyridaben can inhibit or induce autophagy in the placenta, can have adverse effects on early pregnancy, and can cause neurotoxicity and reproductive abnormalities in dopamine neurons in males [6,7]. In addition, pyridaben has been found in many rivers and is highly toxic to aquatic organisms, resulting in severe cardiac malformations and functional abnormalities [8,9]. Owing to its hazardous properties for human health, and its negative influence on nontarget organisms, pyridaben has been labeled as a hazardous substance (Number: 7052) in the Hazardous Substances Data Bank of the National Library of Medicine (USA) [10]. To ensure food safety, maximum residue limits (MRLs) for pyridaben in agro-products have been stipulated in many countries. The national food safety standard of China (GB 2763-2021) has defined the MRLs for pyridaben, in cucumber and tangerine, as 0.1 mg kg^−1^ and 2.0 mg kg^−1^, respectively. The EU has defined the MRLs in cucumber and citrus fruits as 0.15 mg kg^−1^ and 0.3 mg kg^−1^, respectively (EU 2020/1565). The U.S. Environmental Protection Agency (EPA) has also defined the lowest MRL in milk as 0.01 mg kg^−1^. Therefore, the detection of pyridaben, in the environment and in food samples, is of great importance.

To date, several conventional instrumental analysis methods have been reported for pyridaben detection, such as high-performance liquid chromatography (HPLC) [11,12], liquid chromatography coupled with tandem mass spectrometry (LC-MS/MS) [13], ultrahigh-performance LC-MS/MS (UPLC-MS/MS) [14], and gas chromatography (GC) [15,16]. Although these methods are accurate and precise, their disadvantages include being time-consuming and requiring equipment, which limits their application for pyridaben rapid detection [17,18]. Therefore, it is necessary to develop a convenient and simple method for pyridaben on-site detection, to ensure food and environmental safety.

Enzyme-linked immunosorbent assay (ELISA) is regarded as the gold standard technique for rapid analyte detection [19,20]. Nevertheless, the essential washing and incubation steps make it unsuitable for on-site detection. Paper-based competitive lateral flow immunoassay (LFIA) is a state-of-the-art platform used for small-molecule contaminant on-site detection, including pesticide residues [21,22]. In this technique, gold nanoparticles (AuNPs) are the primary reporters because they allow observation of the results, in a straightforward manner, by the naked eye [23,24]. Traditional AuNP-based LFIA is typically performed by visual inspection of test line (T-lines) color changes, which makes it difficult to capture a weak signal change, resulting in insufficient sensitivity and a poor user experience, particularly for untrained personnel [25]. The colorimetric LFIA (CLFIA), that relies on detection of the T-line and control line (C-line), may be an effective platform for addressing the above shortcomings. In the CLFIA, the excellent haptens and antibodies are crucial reagents for pyridaben detection. Miyamoto et al. incorporated a carboxyl or hydroxymethyl group onto the two tert-butyl groups in pyridaben, to afford two carboxyl and two hydroxymethyl compounds as haptens, by a computational chemistry approach. These compounds were used to prepare anti-pyridaben polyclonal antibodies, which showed the lowest 50% inhibitory concentration (IC_50_) of 8.90 ng mL^−1^ for pyridaben in indirect competitive ELISA (ic-ELISA) [26]. Liu et al. prepared anti-pyridaben mAbs, using a novel hapten, by coupling mercaptopropionic acid and pyridaben, and the mAb 3C2 showed the lowest IC_50_ of 2.36 ng mL^−1^ in ic-ELISA and the lowest cut-off value of 25 ng g^−1^ in LFIA [27].

In this study, a pyridaben hapten was synthesized for mAbs preparation. Furthermore, the sensitivities of the prepared mAbs were estimated by using ic-ELISA, and the mAb with the highest sensitivity was selected for the development of a competitive CLFIA. The sensitivity, specificity, and accuracy of the CLFIA were evaluated by pyridaben standard solution, pyridaben analogs, and spiked samples. In addition, the residues of pyridaben in cabbage samples were measured by the CLFIA and HPLC simultaneously, and the obtained results were compared.

## 2. Materials and Methods

### 2.1. Reagents and Apparatus

Standards of pyridaben (99.8%), pyridaphenthion (99.2%), pymetrozine (99.8%), buprofezin (98.4%), thiamethoxam (98.2%), and acetamiprid (99.2%) were provided by the Shanghai Pesticide Research Institute Co., Ltd. (Shanghai, China). Chloroauric acid (HAuCl_4_), Freund’s incomplete and complete adjuvant, bovine serum albumin (BSA), and ovalbumin (OVA) were purchased from Sigma-Aldrich (Milwaukee, WI, USA). N,N′-dicyclohexylcarbodiimide (DCC) and N-hydroxysuccinimide (NHS) were purchased from the Aladdin Industrial Corporation (Shanghai, China). Reagents for cell culture were obtained from ThermoFisher Scientific (Waltham, USA). Nitrocellulose (NC) membranes were purchased from Sirui Science & Technology Co., Ltd. (Wuhan, China). Horseradish peroxidase (HRP)-labeled goat anti-mouse IgG was purchased from Boster (Wuhan, China). The mAb subtype was determined using an IsoQuickTM Kit (Sigma, St. Louis, MO, USA). Animal experiments were approved by the Department of Science and Technology of Jiangsu Province (license number: SYXK (SU) 2021-0086).

The absorbance was measured using a Molecular Devices SpectraMax M5 (San Jose, CA, USA). The morphology of the AuNPs was characterized by transmission electron microscopy (TEM, H-7650, Kitakyushu, Japan). The coating antigen and goat anti-mouse IgG were sprayed on the NC membrane using an XYZ-3000 dispensing platform (Bio-Dot, Irvine, CA, USA) and cut into strips using a CM 4000 guillotine cutter (Bio-Dot, Irvine, USA). Pyridaben was detected using an Agilent 1260 HPLC (Santa Clara, CA, USA).

### 2.2. Preparation of the Pyridaben Hapten and Antigen

**Figure 1:**
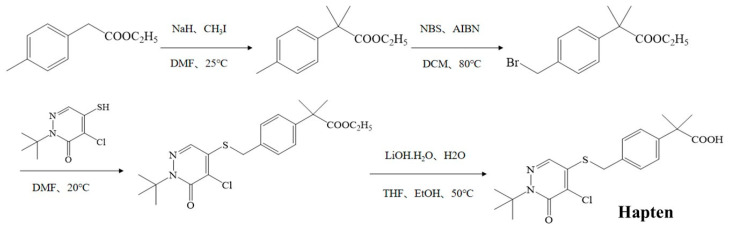
Synthesis route for the pyridaben hapten.

As shown in Figure 1, the pyridaben hapten was synthesized by introducing a carboxyl group at the end of the pyridaben. A total of 60 mL of N,N-dimethylformamide (DMF), containing 5 g of ethyl p-tolylacetate, was added dropwise into 60 mL of DMF containing 3.93 g sodium hydride at 0 °C, and the solution was stirred continuously for 1 h. Then, 7.33 mL of iodomethane was added and reacted at 25 °C for 5 h. The crude product was purified by column chromatography (SiO_2_, petroleum ether/ethyl acetate = 1:0 to 10:1) to obtain ethyl 2-p-methylphenyl-2-methylpropionate (product 1). Then, 2 g of product 1 and 2.07 g of N-bromosuccinimide, dissolved in 20 mL of dichloromethane, were mixed with 0.08 g of azodiisobutyronitrile and stirred for 1 h at 80 °C. The ethyl 2-(4-(bromomethyl) phenyl)-2-methylpropionate (product 2) was obtained after the purification method of product 1. The product 2 (1.2 g), 0.92 g of 2-*tert*-butyl-4-chloro-5-mercapto-2h-pyridazine-3-one and 0.67 g of K_2_CO_3_ were dissolved in 20 mL of DMF, and the mixture was stirred for 4 h at 20 °C. After purification, ethyl-2-(4-(((1-(*tert*-butyl)-5-chloro-6-oxo-1,6-dihydropyridazin-4-yl)thio)methyl)phenyl)-2-methylpropanoate (product 3) was obtained. The product 3 (1.2 g) dissolved in 4 mL of EtOH and 4 mL of tetrahydrofuran was mixed with 0.48 g of LiOH·H_2_O in 4 mL of pure water and stirred for 4 h at 50 °C. The pyridaben hapten was obtained after purification (SiO_2_, petroleum ether/ethyl acetate = 100:0 to 10:1). The obtained hapten was further identified by mass spectrometry (*m*/*z*) and ^1^H NMR.

The pyridaben hapten was conjugated with BSA (hapten-BSA) and OVA (hapten-OVA) using the active ester method to prepare the immunogen and the coating antigen, respectively. In brief, 0.1 mmol of pyridaben hapten, 0.1 mmol of DCC, and 0.1 mmol of NHS were dissolved in 2.5 mL of DMF and stirred overnight in the dark. After centrifugation, the supernatant was collected and equally added into 5 mL of phosphate-buffered saline (PBS) containing 10 mg mL^−1^ BSA or OVA. The mixture was stirred for 4 h at room temperature (RT) and the obtained conjugates were dialyzed, split, and stored at −20 °C for further use. The coupling ratios of the carrier and hapten were calculated according to the following formula: coupling ratio = (*ε* (conjugate) − *ε* (BSA or OVA)/*ε* (hapten) (*ε* represents molar absorption coefficient) [17].

### 2.3. Preparation and Characterization of MAbs

The female BALB/c mice (6–8 weeks) were used for immunity. One hundred microliters of immunogen (1 mg mL^−1^) and isochoric Freund’s complete adjuvant were emulsified and used for initial immunization. Three weeks later, the same amount of immunogen, and Freund’s incomplete adjuvant, were utilized for the subsequent immunizations at two week intervals, until the titer of the serum was held on a platform. The mouse that produced the highest titer and sensitivity was selected for booster immunization and cell fusion. The positive cells were chosen, subcloned, and cultured for ascites preparation. The anti-pyridaben mAbs were purified using staphylococcal protein A column, and their sensitivities were evaluated by ic-ELISA. In addition, their specificities were assessed in terms of the cross-reactivities (CRs) with structural analogs of pyridaben, including pyridaphenthion, pymetrozine, buprofezin, thiamethoxam, and acetamiprid. The CRs were defined as follows: CRs (%) = [IC_50_ (pyridaben)/IC_50_ (analogs)] × 100.

### 2.4. Preparation of AuNP Labeled MAb

AuNPs of approximately 25 nm were synthesized using the sodium citrate as a reduction agent. The AuNP-labeled mAb (AuNP-mAb) was prepared according to the reported method [28], where the minimum dosage of mAb was determined via a salt precipitation test. Briefly, 2.6 mg of mAb was added to 50 mL of AuNP solution (pH 8.2) with gentle shaking for 1 h. Then, the remaining active sites were passivated by 5 mL of 10% BSA (*w/v*) for an additional duration of 1 h. The free mAb was removed by centrifugating at 8000 rpm for 15 min, and the AuNP-mAb was concentrated to 5 mL in borate buffer (10 mM, containing 2% BSA and 3% sucrose). The prepared AuNPs and the AuNP-mAb were characterized by TEM (Figure S1A) and ultraviolet visible (UV-vis) spectroscopy (Figure S1B).

### 2.5. Assembly of the CLFIA

The CLFIA test strips contain a sample pad, a conjugate pad, an NC membrane, an absorbent pad and a PVC plate. The 0.3 mg mL^−1^ coating antigen and the 0.125 mg mL^−1^ goat anti-mouse IgG antibody, dispersed on an NC membrane with a speed of 1µL cm^−1^, were served as T-line and C-line. A conjugate pad was immobilized with the AuNP-mAb. After drying for 2 h at 37 °C, they were assembled and cut into 4 mm wide individual test strips. Finally, the strips were put into a plastic shell, vacuum-packed independently and stored at RT for further use.

### 2.6. Protocol of the CLFIA

One hundred microliters of pyridaben standard, or sample solution (containing 20% acetonitrile), were dropped on the sample pad and allowed to flow across the NC membrane by capillary action. The test results could be interpreted quickly by the naked eye, within 10 min, according to the colorimetric signal of the T/C-line.

### 2.7. Optimization of the CLFIA

The performances of immunoassays are significant influenced by the reaction conditions. Therefore, the coating antigen (0.075, 0.15, 0.3, 0.6, 1.2 mg mL^−1^), AuNP-mAb (1, 2, 3, 4, 5, 6 µL), and goat anti-mouse IgG (0.075, 0.1, 0.125, 0.15, 0.175 mg mL^−1^) were optimized to improve the performance of the CLFIA, where higher sensitivity was selected as the optimal condition. In addition, the working buffer parameters, including organic solvent type (methanol, acetonitrile, acetone) and content (5%, 10%, 15%, 20%, 25%), concentration of Na^+^ (0.07, 0.14, 0.28, 0.56, 1.12 M), pH (5.0, 6.0, 7.4, 8.0, 9.0), and the contents of Tween-20 (0.05%, 0.1%, 0.2%, 0.4%, 0.8%, 1.6%) and PEG-20000 (0.05%, 0.1%, 0.2%, 0.4%, 0.8%), were sequentially studied to improve the sensitivity of the CLFIA.

### 2.8. Analysis of the Spiked Samples

The environmental sample of paddy water was obtained from a farm, and the agro-products of cucumber, cabbage, tangerine, and orange were purchased from a local supermarket in Zhengzhou. All the samples were homogenized, confirmed to be pyridaben-free by HPLC, divided into 3 parts and stored at −20 °C. The accuracy of immunoassays should be evaluated in at least 4 samples, and each sample with 3 concentration levels (low, medium and high), according to the International Union of Pure and Applied Chemistry (IUPAC) reports on pesticides. In our study, pyridaben in 1 mL of acetonitrile was added to 5 g of homogenized samples, and the final concentrations of pyridaben were 1.25, 2.5, 5 and 10 ng mL^−1^ for paddy water; 12.5, 25, 50 and 100 ng g^−1^ for cucumber; and 25, 50, 100 and 200 ng g^−1^ for cabbage, tangerine, and orange. The paddy water was analyzed directly after dilution. The other solid samples were extracted with 5 mL of PBS containing 40% acetonitrile. After vortexing for 5 min, the samples were centrifuged for 5 min at 4000 rpm. The supernatant was collected and used for the CLFIA analysis after appropriate dilution.

### 2.9. HPLC Analysis and Validation

Fifty blind cabbage samples, named S1−S50, were prepared, by nontesting personnel, by randomly spiking with standard pyridaben solutions. The amount of pyridaben was analyzed simultaneously by means of the CLFIA and HPLC. The pretreatment for the CLFIA was the same as the spiked samples. For HPLC, 10 g of homogenized cabbage samples were extracted using 10 mL of acetonitrile, with shaking for 5 min at 2500 rpm and ultrasonication for 10 min. The organic and aqueous phases were separated by adding 2 g of NaCl and shaking for another 10 min. The mixture was centrifuged at 4000 rpm for 10 min, and 1 mL of supernatant was transferred, dried and redissolved in 1 mL of mobile phase (acetonitrile/0.1% formic acid-water (85:15, *v/v*)) for HPLC detection with an SB-C18 column (250 mm × 4.6 mm, 5 μm). The flow rate, injection volume, and detection wavelength were 1 mL min^−1^, 20 μL and 240 nm, respectively.

## 3. Results and Discussion

### 3.1. Identification of Hapten and Antigen

The measured *m*/*z* and ^1^H NMR results (Figure S2) for pyridaben hapten were as follows: ESI-MS, *m*/*z*, 395.13 [M + H]^+^; ^1^H NMR (400 MHz, DMSO-d6) δ 8.054 (s, 1H), 7.366–7.327 (q, J = 8.4 Hz, 4H), 4.501 (s, 2H), 1.563 (s, 9H), 1.409 (s, 6H). These results indicated that the hapten was synthesized successfully.

The UV-vis spectra of hapten, BSA, OVA, immunogen, and coating antigen are shown in Figure S3. The immunogen showed an obvious redshift and blueshift, compared to BSA and hapten, respectively (Figure S3A), while the coating antigen showed a blueshift, compared to OVA and hapten (Figure S3B). The shift of the plasma resonance bands illustrated that the antigens were prepared successfully. In addition, the coupling ratios of the immunogen and the coating antigen were estimated to be 15:1 and 10:1, respectively.

### 3.2. Characterization of MAbs

**Figure 2:**
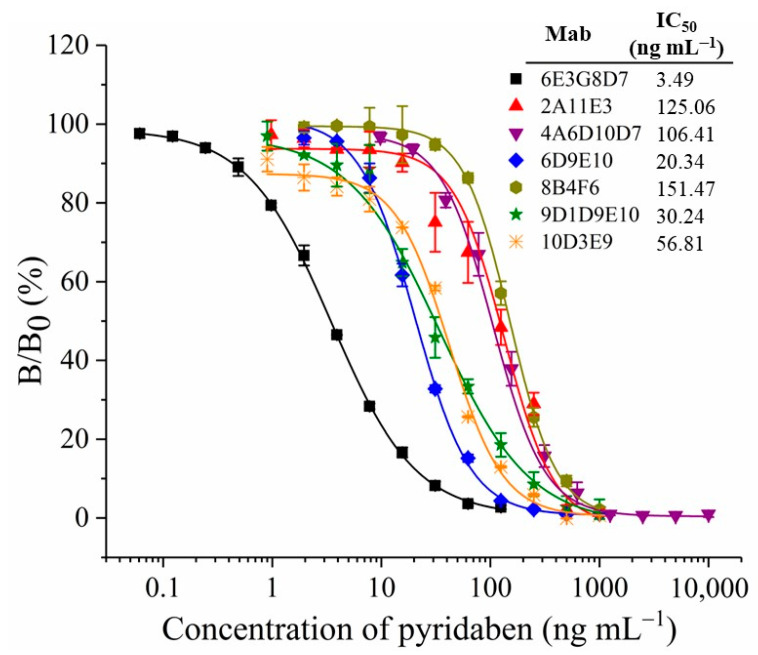
Standard curves of ic-ELISA with different mAbs.

The titers and sensitivities of tail blood from the five mice were measured by ELISAs after five immunizations, and the concentration of the coating antigen was 10 µg mL^−1^. All five mice showed higher titers and sensitivities (Table S1). The highest titer was observed for mouse No. 3 at 1:64,000, while mouse No. 4 showed the highest sensitivity, with an inhibition ratio (1 µg mL^−1^ of pyridaben standard) of 77.9%. Mouse No. 4 underwent cell fusion, and the positive hybridoma clones, 6E3G8D7, 2A11E3, 4A6D10D7, 6D9E10, 8B4F6, 9D1D9E10, and 10D3E9, that can stably produce anti-pyridaben mAbs, were obtained. As shown in Figure 2, the standard curves of ic-ELISA were established by plotting the binding rate (B/B_0_) against the concentration of pyridaben. The mAb, 6E3G8D7, showed the highest sensitivity, with an IC_50_ value of 3.49 ng mL^−1^. In addition, the CR values were less than 0.1% for structural analogs of pyridaben (Table S2), which indicated the high specificity. Therefore, mAb 6E3G8D7, which was identified as the IgG2b subtype with an affinity of 9.49 × 10^9^ L/mol, was selected for future CLFIA.

### 3.3. Generation of the Colorimetric Signal on the CLFIA

**Figure 3:**
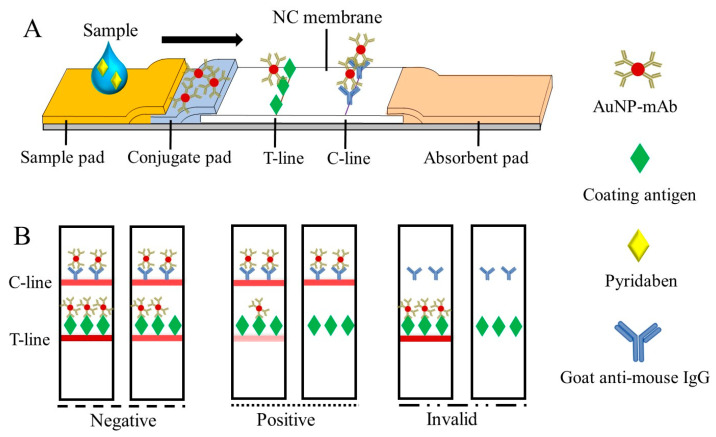
The schematic of the CLFIA (**A**) and judgment of the test results (**B**).

When the test sample was added to the sample pad, the fixed AuNP-mAb was redissolved and moved upward, along with the NC membrane, to generate visual signals on the T-line and C-line (Figure 3A), which can be read by the naked eye within 10 min. As shown in Figure 3B, the test results were divided into: (1) negative (–), where the samples were pyridaben-free or less than the limit of detection (LOD), and the signal intensity on the T-line was greater than or similar to that of the C-line; (2) positive (+), in which the pyridaben was above the LOD or inhibited the immune reaction completely, and the signal intensity on the T-line was weaker than that on the C-line; and (3) invalid, where the C-line was colorless.

### 3.4. CLFIA Optimization

The concentrations of mAbs and antigens are important for the sensitivity of the CLFIA [29,30]. In our study, AuNP-mAb (1 to 6 µL) and coating antigen (0.075 to 1.2 mg mL^−1^) were used to detect pyridaben standard (0 to 80 ng mL^−1^) in PBS, with 0.1 mg mL^−1^ goat anti-mouse IgG fixed on the C-line. As shown in Table S3, the sensitivity was low, and the test results for negative samples were false-positive when the coating antigen concentration was less than 0.3 mg mL^−1^. The test results were accurate, and the highest sensitivity was achieved when the coating antigen concentration was 0.3 mg mL^−1^ and the AuNP-mAb volume was 4 μL. Thus, these values were selected as the optimal conditions for the CLFIA. The concentration of goat anti-mouse IgG (0.075 to 0.175 mg mL^−1^) was further optimized. As shown in Table S4, 0.125 mg mL^−1^ was selected according to achievement of the sensitivity and accuracy required for negative samples. Using the same evaluation criteria, the optimal conditions of the working buffer were determined to be 0.4% Tween-20, 0.14 M Na^+^, pH 7.4, 0.2% PEG 20000, and 20% acetonitrile (Table S5).

### 3.5. Sensitivity and Selectivity of the CLFIA

**Figure 4:**
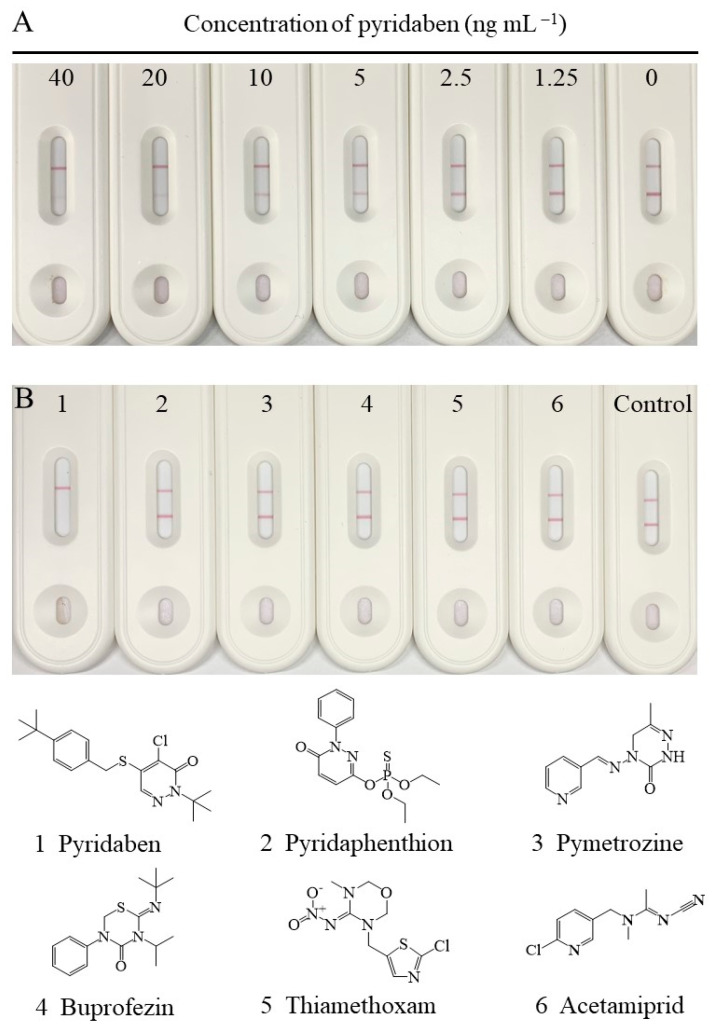
(**A**) The standard curve of the CLFIA for pyridaben. (**B**) The selectivity of the CLFIA for pyridaben.

Under the abovementioned optimal conditions, the changes in signal intensity, with different concentrations of pyridaben standard, are shown in Figure 4A. The signal intensity on the T-line decreased with increasing concentrations of pyridaben, while the C-line increased. The signal intensity on the T-line was greater than or similar to that on the C-line when the concentration of pyridaben was less than 5 ng mL^−1^. Thus, 5 ng mL^−1^ could be defined as the visual LOD of the CLFIA, which was 8 times higher than the traditional LFIA, according to the T-line only in Figure 4A. In addition, the colorimetric readout is more convenient for qualitative and semiquantitative analysis by the naked eye than traditional AuNP-based LFIAs.

In a comparison of the immunoassays for pyridaben (Table S6), the proposed CLFIA shows sensitivity 5 times higher than the reported LFIA, according to the LOD, which can be attributed to the colorimetric method of T/C-line [27]. As for ELISA, the proposed CLFIA has significant advantages in response time, on-site testing, and stability, because the CLFIA can obtain the test results within 10 min, by naked eye, after simple pretreatment, and the AuNPs—as probes—are more stable than natural enzymes [26,31].

The selectivity of the CLFIA was assessed by detection of pyridaben analogs (10,000 ng mL^−1^), including pyridaphenthion, pymetrozine, buprofezin, thiamethoxam, and acetamiprid. As shown in Figure 4B, the test result for pyridaben (100 ng mL^−1^) was positive (+), while those of the other analogs were negative (–). These results indicated that the CLFIA was highly specific for pyridaben, which was consistent with the CRs for ic-ELISA based on mAb 6E3G8D7.

### 3.6. Detection of the Spiked Samples

**Figure 5:**
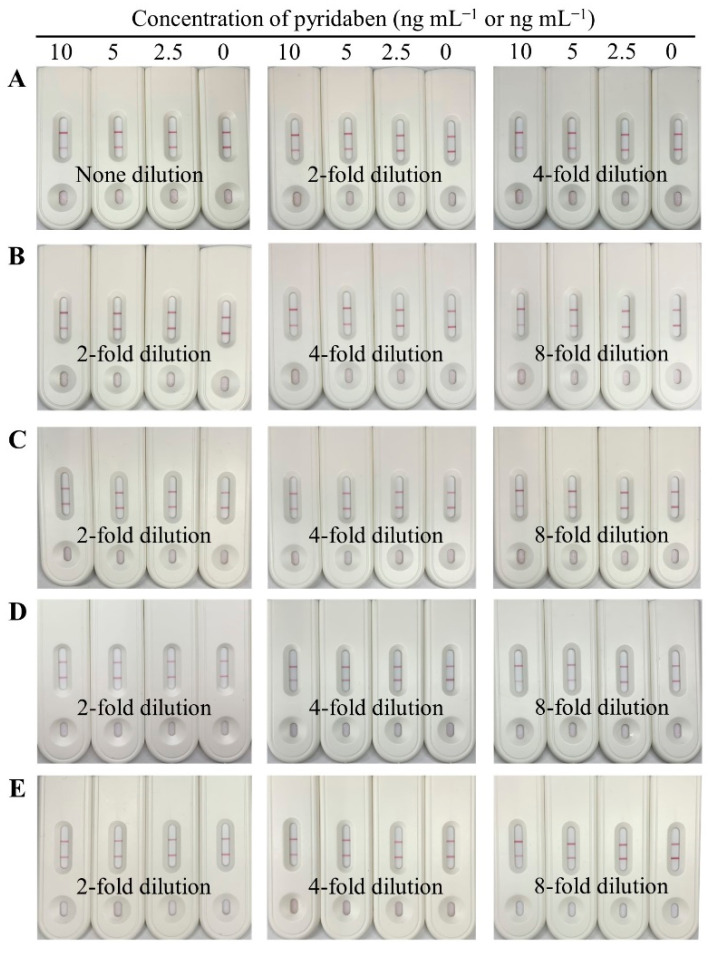
Images of the CLFIA for pyridaben detection in different sample matrices with dilution, from top to bottom, were paddy water (**A**); cucumber (**B**); cabbage (**C**); tangerine (**D**); and orange (**E**).

Matrix interference of sample extracts is a critical factor affecting the accuracy and sensitivity of immunoassays, which can be eliminated by dilution. In our study, the blank paddy water and agro-product extracts were serially diluted with the optimal working buffer containing pyridaben standard, and the final concentrations were 0, 2.5, 5, and 10 ng mL^−1^ or ng g^−1^. As shown in Figure 5, no dilution for paddy water, 4-fold dilution for cucumber, and 8-fold dilution for cabbage, tangerine, and orange were selected, because the detection results of 5 ng mL^−1^ were consistent with the results for the working buffer, which indicated that the matrix interferences were negligible.

Under the above dilutions, the detection results of the CLFIA, for the spiked samples, were negative (−) when the concentrations of pyridaben were 2.5 ng mL^−1^ for paddy water; 12.5 ng g^−1^ for cucumber; and 25 ng g^−1^ for cabbage, tangerine, and orange (Table 1). On the other hand, the results were positive (+) when the concentration was greater than or equal to 5 ng mL^−1^ for paddy water; 25 ng g^−1^ for cucumber; and 50 ng g^−1^ for cabbage, tangerine, and orange. These results indicated that the visual LODs of the CLFIA for paddy water were 5 ng mL^−1^; 25 ng g^−1^ for cucumber; and 50 ng g^−1^ for cabbage, tangerine, and orange, which satisfies the requirements for pyridaben detection, according to the MRLs, such as 0.1 mg kg^−1^ for cucumber and 2.0 mg kg^−1^ for fruits in China, 0.15 mg kg^−1^ for cucumber and 0.3 mg kg^−1^ for citrus fruits in the EU, and 0.5 mg kg^−1^ for cucumber in the USA.

### 3.7. Validation with HPLC

The pyridaben residues of the 50 cabbage samples were simultaneously detected by the CLFIA and HPLC to verify their practicability. The chromatogram of the pyridaben standard is shown in Figure S4A. The standard curve of pyridaben in the matrix buffer was y = 598.83x − 4.9964 (R^2^ = 0.9997), which was consistent with the standard curve in solvent (y = 530.39x + 6.6925 (R^2^ = 0.9994)) (Figure S4B). In addition, the slope ratio of 1.13 (598.83/530.39) indicated that the matrix interferences were acceptable (the generally acceptable range is 0.8–1.2) [32]. Thus, the standard curve in the solvent was employed to quantify pyridaben in cabbage samples, and the limit of quantification (LOQ) was 0.02 mg kg^−1^, according to a 10-fold signal/noise ratio.

As shown in Table 2, the CLFIA detection results showed that 25 of the 50 cabbage samples tested positive (+) (pyridaben residues greater than 0.05 mg kg^−1^), and the results for other samples were negative (−), which was consistent with the HPLC results. These results indicated that CLFIA are accurate and reliable for pyridaben on-site detection.

## 4. Conclusions

In this study, pyridaben hapten was synthesized and used to generate seven anti-pyridaben mAbs. The mAb, 6E3G8D7, which showed the best sensitivity and no CRs to the analogs of pyridaben in ic-ELISA, was further utilized to develop a competitive CLFIA for pyridaben detection. The CLFIA showed excellent sensitivity and specificity for pyridaben, and the accuracy confirmed by HPLC indicated that the CLFIA could be used for pyridaben screening in agro-products and environmental samples. The detection results can be judged directly within 10 min, by the naked eye, without any instrument. In addition, the CLFIA could be used for pyridaben on-site detection, and the visual LODs satisfy the requirements of the MRLs in China, the EU, and the USA. Compared with traditional LFIA, the developed CLFIA is more sensitive and convenient for the qualitative and semiquantitative detection of analytes than the signal changes of the T-line only. Such improvement in the CLFIA is expected to promote the next-generation rapid and sensitive monitoring of pesticide residues, thus providing improved outcomes.

## Figures and Tables

**Table 1:** The detection results in spiked samples by the CLFIA.

Sample	Spiked (ng g^−1^ or ng mL^−1^)	Results of CLFIA ^a^			MRL (ng g^−1^ or ng mL^−1^)
		1	2	3	
Paddy water	0	−	−	−	10 ^b^
	1.25	−	−	−	
	2.5	−	−	−	
	5	+	+	+	
	10	+	+	+	
Cucumber	0	−	−	−	100
	12.5	−	−	−	
	25	+	+	+	
	50	+	+	+	
	100	+	+	+	
Cabbage	0	−	−	−	2000
	25	−	−	−	
	50	+	+	+	
	100	+	+	+	
	200	+	+	+	
Tangerine	0	−	−	−	2000
	25	−	−	−	
	50	+	+	+	
	100	+	+	+	
	200	+	+	+	
Orange	0	−	−	−	2000
	25	−	−	−	
	50	+	+	+	
	100	+	+	+	
	200	+	+	+	

^a^ “+” represents positive results and “−” represents negative results ^b^ the value of paddy water is referenced the MRLs for milk in USA, EU and Japan.

**Table 2:** The detected results for cabbage samples by CLFIA and HPLC.

Sample Number	Results		Sample Number	Results	
	HPLC (mg kg^−1^)	CLFIA ^a^		HPLC (mg kg ^−1^)	CLFIA ^a^
S1	0.32	+	S26	0.21	+
S2	0.46	+	S27	<LOQ	−
S3	0.15	+	S28	0.48	+
S4	0.12	+	S29	<LOQ	−
S5	0.09	+	S30	0.08	+
S6	0.11	+	S31	<LOQ	−
S7	<LOQ	−	S32	<LOQ	−
S8	<LOQ	−	S33	0.27	+
S9	<LOQ	−	S34	<LOQ	−
S10	<LOQ	−	S35	<LOQ	−
S11	0.35	+	S36	<LOQ	−
S12	0.08	+	S37	0.11	+
S13	0.13	+	S38	<LOQ	−
S14	0.06	+	S39	<LOQ	−
S15	<LOQ	−	S40	0.09	+
S16	0.58	+	S41	<LOQ	−
S17	<LOQ	−	S42	<LOQ	−
S18	<LOQ	−	S43	0.13	+
S19	0.28	+	S44	0.16	+
S20	0.14	+	S45	<LOQ	−
S21	<LOQ	−	S46	0.11	+
S22	<LOQ	−	S47	<LOQ	−
S23	<LOQ	−	S48	0.07	+
S24	0.05	+	S49	0.28	+
S25	<LOQ	−	S50	<LOQ	−

^a^ “+” represents positive detection results and “−” represents negative detection results.

## Data Availability

The data presented in this study are available on request from the corresponding author.

## Supplementary Materials

The following supporting information can be downloaded at: https://www.mdpi.com/article/10.3390/bios13050545/s1, Figure S1: the TEM image of AuNPs (A) and UV-vis spectrums of AuNPs and AuNP-mAb (B); Figure S2: the 1H NMR for pyridaben hapten; Figure S3: the UV spectra of hapten, carrier proteins, and their conjugates; Figure S4: chromatogram of pyridaben (A) and standard curves in matrix (B); Table S1: the titers and sensitivities of serums; Table S2: CRs of ic-ELISA for the analogs of pyridaben (n = 3); Table S3: the detection results of theCLFIA with different combinations of coating antigen and AuNP-mAb; Table S4: the detection results of the CLFIA with different concentrations of goat anti-mouse IgG; Table S5: the optimal parameters of working buffer for the CLFIA; Table S6: comparison of the proposed CLFIA with other previous immunoassays for pyridaben detection.
