# Supplementary material for: Monoclonal Antibody-Based Colorimetric Lateral Flow Immunoassay for the Detection of Pyridaben in the Environment
Source: Biosensors (Basel). 2023 May 13;13(5):545. doi: 10.3390/bios13050545 (PMC10216005; doi:10.3390/bios13050545)
Supplement: Supplementary file 1 [file biosensors-13-00545-s001.zip › biosensors-2382488-supplementary.pdf]

biosensors

MDPI

Supporting Information

Monoclonal Antibody-Based Colorimetric Lateral Flow Immunoassay for the Detection of Pyridaben in the Environment

He Chen [1,2,†], Hao Liu [3,†], Yanran Ji [4], Zekun Sha [4], Li An [1,2], Meng Li [1,2], Di Zhang [1,2], Xujin Wu [1,2,*] and Xiude Hua [4,*]

[1] Institute of Quality Standard and Testing Technology for Agro-Products, Henan Academy of Agricultural Sciences, Zhengzhou 450002, China
[2] Key Laboratory of Grain Quality and Safety and Testing Henan Province, Zhengzhou 450002, China
[3] School of Life Sciences, Henan University, Kaifeng 475004, China
[4] College of Plant Protection, Nanjing Agricultural University, Nanjing 210095, China
* Correspondence: xujinwu2005@126.com (X.W.); huaxiude@njau.edu.cn (X.H.)
† These authors contributed equally to this work.

Table S1. The titers and sensitivities of serums.

Number of Serum	Titer	Sensitivity[a]
1	1:32000	59.7 %
2	1:32000	66.6%
3	1:64000	67.3%
4	1:32000	77.9%
5	1:16000	75.0%

[a] The sensitivity represents the inhibition ratio of 1 μg mL^{-1} pyridaben standard.

Table S2. CRs of ic-ELISA for the analogs of pyridaben (n = 3).

Compounds	Structure	IC_{50} (ng mL^{-1})	CR (%)
Pyridaben	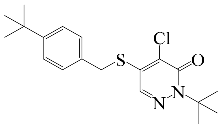	3.49	100
Pyridaphenthion	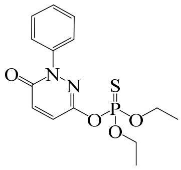	>10000	<0.1
Pymetrozine	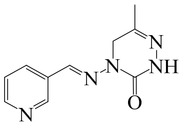	>10000	<0.1
Buprofezin	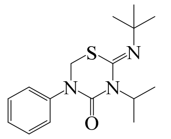	>10000	<0.1

Thiamethoxam	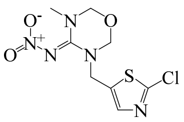	>10000	<0.1
Acetamiprid	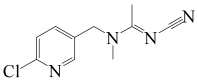	>10000	<0.1

Table S3. The detection results of CLFIA with different combinations of coating antigen and AuNP-mAb.

Coating Antigen (mg mL^{-1})	AuNP-mAb (μL)	Concentration of Pyridaben (ng mL^{-1})			
		0	20	40	80
1.2	1	−	−	−	+
	2	−	−	−	+
	3	−	−	−	+
	4	−	−	−	+
	5	−	−	−	+
	6	−	−	−	+
0.6	1	+	+	+	+
	2	−	−	−	+
	3	−	−	−	+
	4	−	−	−	+
	5	−	−	−	+
	6	−	−	−	+
0.3	1	+	+	+	+
	2	+	+	+	+
	3	+	+	+	+
	4	−	−	+	+
	5	−	−	+	+
	6	−	−	+	+
0.15	1	+	+	+	+
	2	+	+	+	+
	3	+	+	+	+
	4	+	+	+	+
	5	+	+	+	+
	6	+	+	+	+
0.075	1	+	+	+	+
	2	+	+	+	+
	3	+	+	+	+
	4	+	+	+	+
	5	+	+	+	+
	6	+	+	+	+

Notes: (+) represents positive result and (−) represents negative result.

Table S4. The detection results of CLFIA with different concentration of goat anti-mouse IgG.

Goat Anti-Mouse IgG (mg mL^{-1})	Concentration of Pyridaben (ng mL^{-1})			
	0	20	40	80
0.075	−	−	−	−
0.1	−	−	−	+
0.125	−	−	+	+
0.15	+	+	+	+
0.175	+	+	+	+

Notes: (+) represents positive result; (−) represents negative result.

Table S5. The optimal parameters of working buffer for the CLFIA.

Working Buffer		Concentration of Pyridaben (ng mL⁻¹)				
		0	2.5	5	10	20
Tween-20 (%)	0.05	−	−	−	−	−
	0.1	−	−	−	−	+
	0.2	−	−	−	−	+
	0.4	−	−	−	+	+
	0.8	−	−	−	+	+
	1.6	+	+	+	+	+
Na^+ (M)	0.07	−	−	−	−	−
	0.14	−	−	−	+	+
	0.28	−	−	−	+	+
	0.56	−	−	−	+	+
	1.12	+	+	+	+	+
pH	5	−	−	−	−	+
	6	−	−	−	−	+
	7.4	−	−	−	+	+
	8	−	−	−	+	+
	9	−	−	−	+	+
PEG 20000 (%)	0.05	−	−	−	+	+
	0.1	−	−	−	+	+
	0.2	−	−	+	+	+
	0.4	−	−	+	+	+
	0.8	−	−	+	+	+
Methanol （%）	5	−	−	−	+	+
	10	−	−	−	+	+
	15	−	−	−	+	+
	20	−	−	−	−	+
	25	−	−	−	−	+
Acetonitrile （%）	5	−	−	+	+	+
	10	−	−	+	+	+
	15	−	−	+	+	+
	20	−	−	+	+	+
	25	+	+	+	+	+
Acetone （%）	5	−	−	−	+	+
	10	−	−	−	+	+
	15	−	−	−	+	+
	20	−	−	−	+	+
	25	−	−	−	−	+

Notes: "+" represents positive detection result and "−" represents negative detection result.

Table S6. Comparison of the proposed CLFIA with other previous immunoassays for pyridaben detection.

Methods	Probes	LOD (ng mL^{-1})	Response Time (min)	Step	Signal Readout	Ref
ELISA	HRP	–	495[a]	6	Absorption at 405 nm	[26]
LFIA	AuNPs	25	10	1	Signal intensity of T-line only	[27]
CLFIA	AuNPs	5	10	1	Signal intensity of T/C-line	This work

Notes: "–" represents the date not shown; [a] the incubation for overnight was replaced by incubation for 2 h.

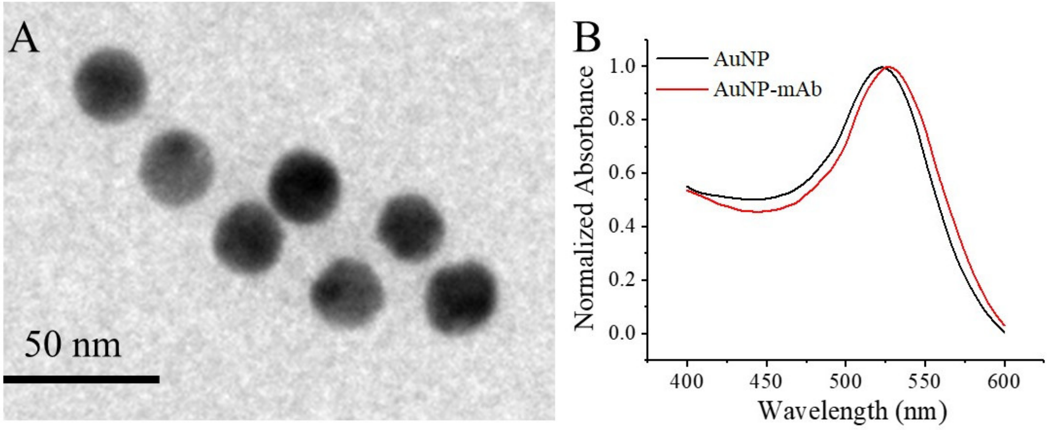

Figure S1. The TEM image of AuNPs (**A**) and UV-vis spectrums of AuNPs and AuNP-mAb (**B**).

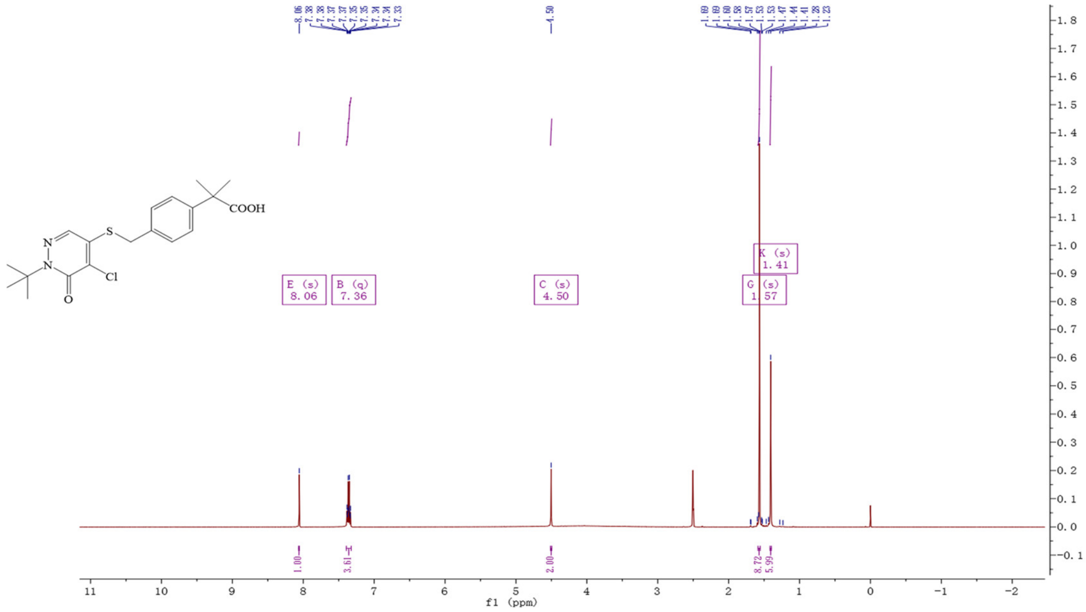

Figure S2. The ^{1}H NMR for pyridaben hapten.

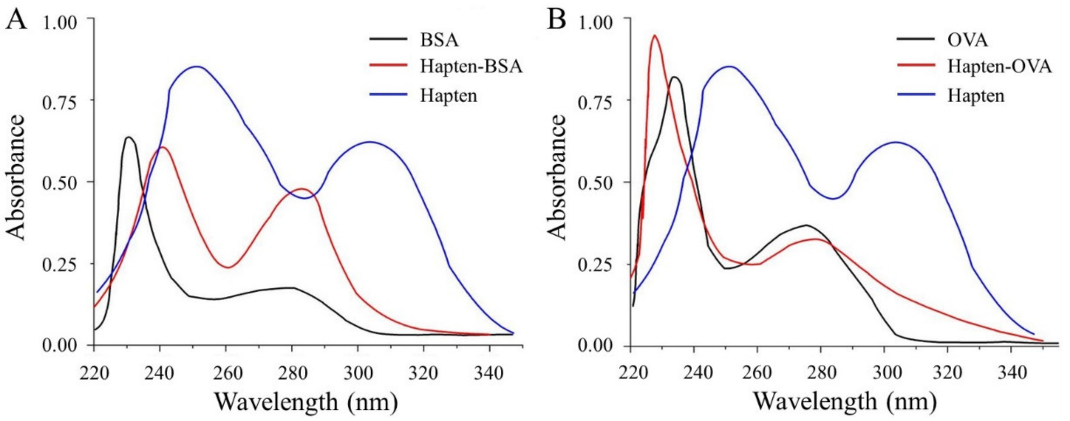

Figure S3. The UV spectra of hapten, carrier proteins and their conjugates.

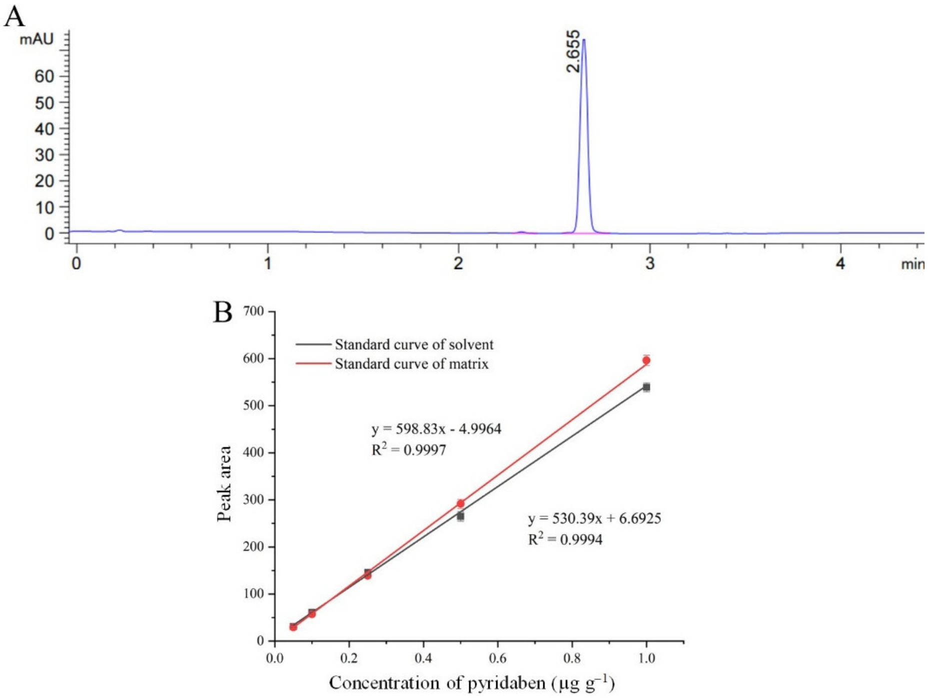

Figure S4. Chromatogram of pyridaben (**A**) and standard curves in matrix (**B**).
